# Google trend analysis of the Indian population reveals a panel of seasonally sensitive comorbid symptoms with implications for monitoring the seasonally sensitive human population

**DOI:** 10.1186/s12963-024-00349-7

**Published:** 2024-12-30

**Authors:** Urmila Gahlot, Yogendra Kumar Sharma, Jaichand Patel, Sugadev Ragumani

**Affiliations:** Bioinformatics Group, Defense Institute of Physiology and Allied Sciences, Defense Research and Development Organization, Lucknow Road, Timarpur, Delhi India

**Keywords:** Asthma, Obesity, Pulmonary hypertension, Pulmonary fibrosis, Seasonality, Seasonally sensitive population

## Abstract

Seasonal variations in the environment induce observable changes in the human physiological system and manifest as various clinical symptoms in a specific human population. Our earlier studies predicted four global severe seasonal sensitive comorbid lifestyle diseases (SCLDs), namely, asthma, obesity, hypertension, and fibrosis. Our studies further indicated that the SCLD category of the human population may be maladapted or unacclimatized to seasonal changes. The current study aimed to explore the major seasonal symptoms associated with SCLD and evaluate their seasonal linkages via Google Trends (GT). We used the Human Disease Symptom Network (HSDN) to dissect common symptoms of SCLD. We then exploited medical databases and medical literature resources in consultation with medical practitioners to narrow down the clinical symptoms associated with four SCLDs, namely, pulmonary hypertension, pulmonary fibrosis, asthma, and obesity. Our study revealed a strong association of 12 clinical symptoms with SCLD. Each clinical symptom was further subjected to GT analysis to address its seasonal linkage. The GT search was carried out in the Indian population for the period from January 2015–December 2019. In the GT analysis, 11 clinical symptoms were strongly associated with Indian seasonal changes, with the exception of hypergammaglobulinemia, due to the lack of GT data in the Indian population. These 11 symptoms also presented sudden increases or decreases in search volume during the two major Indian seasonal transition months, namely, March and November. Moreover, in addition to SCLD, several seasonally associated clinical disorders share most of these 12 symptoms. In this regard, we named these 12 symptoms the “seasonal sensitive comorbid symptoms (SSC)” of the human population. Further clinical studies are needed to verify the utility of these symptoms in screening seasonally maladapted human populations. We also warrant that clinicians and researcher be well aware of the limitations and pitfalls of GT before correlating the clinical outcome of SSC symptoms with GT.

**Supplementary Information:**

The online version contains supplementary material available at 10.1186/s12963-024-00349-7.

## Introduction

Humans began to take seasonal exposure for granted, at least in a biological sense. Most of us support positive “adaptations” to seasons. No response to a changing season leads to increased vulnerability to seasonal change and can be considered maladaptive. Most researchers believe the shift between seasonal adaptation and maladaptation can be subtle and rapid. However, in the case of an ineffective adaptive target population, the shift is irreversibly more vulnerable to seasonal changes. Recently, globalization and rapidly changing human lifestyle patterns have had significant impacts on human seasonal adaptation [1, 27]. Although seasonality is a well-known phenomenon in the epidemiology of many lifestyle diseases, analytical tools for examining, evaluating, and comparing seasonal symptoms in diseases are highly limited. The significant association between season and lifestyle diseases has not been explored due to the lack of scientific knowledge and tools to distinguish the seasonally maladapted population from the normal population.

In this direction, through our semantic similarity disease network-based clustering studies, we could narrow down to four human lifestyle disorders, namely, pulmonary hypertension, pulmonary fibrosis, asthma, and obesity, and verify their significant seasonal associations across the globe through Google Trends (GTs) [30]. We also reported these four disorders as seasonal (sensitive) comorbid lifestyle diseases (SCLDs). The so-called “severely seasonally sensitive” human population is expected to be more prone to major seasonal variations in these four SCLDs. Several clinical studies worldwide have reported similar observations on SCLD in terms of our outcomes [5, 15, 20]. Our further studies on SCLD in the four climatic zones of India strengthened our prediction that SCLD is significantly associated with the season [31]. Recent clinical studies have revealed an alarming trend in the prevalence of SCLD, indicating a growing health crisis worldwide as well as in India [7, 29] [23]. To study seasonally sensitive populations in the clinic, SCLD-associated symptoms and their severity during different seasons should be collected from patient’s medical records for at least three years. To our knowledge, no such clinical records are available and publicly accessible for a larger population to address this issue. Considering its importance and limitations, the current study has the following research objectives: (A) To collect the SCLD clinical symptoms strongly associated with pulmonary hypertension, pulmonary fibrosis, asthma, and obesity through a human disease symptom network (HSDN), clinical literature and public databases in consultation with medical practitioners, and (B) to verify the seasonal associations of symptoms using GT in the Indian population.

Our GT study and literature support revealed 12 symptoms strongly associated with seasonal changes, most of which exhibited sudden increases or decreases in search volume during the major Indian seasonal transition months, namely, March and November. Several clinical studies have shown that most of these common symptoms coexist with or are mostly comorbid with several known seasonal disorders. We named these 12 common seasonal symptoms “season sensitive comorbid” (SSC) symptoms of the human population. These SSC symptoms could be readily exploited to clinically study their severity during seasonal variations to address the so-called seasonally maladapted “seasonally sensitive population”.

## Methodology

### SCLD-associated symptoms

The clinical note is a significant part of patient records in an unstructured free-text format. However, extracting symptoms from the corpus is difficult because of their variety and syntactic ambiguity [38]. Instead, to identify symptom entities accurately, we relied on symptom‒disease associations from the Mala Cards [32, 33]Human Symptoms Disease Network (HSDN) (Zhou et al. 2014a), Unified Medical Language System (UMLS) databases [10, 18] Human Phenotype Ontology (HPO) [34], and Medical Subject Headings (MeSH) [47] (Supplementary S1). We used the International Classification of Diseases, 10th revision (ICD-10-CM) for the identification of symptoms associated with four SCLD diseases to avoid ambiguity and multiple conventions of the symptom terminologies. According to the National Institutes of Health (NIH) and the American Obesity Society (AOS), obesity is treated as a complex disease rather than a symptom. However, owing to insufficient data, the American Medical Association (AMA) considers obesity a symptom instead of a disease [36].

#### Selection of SCLD symptoms through HSDN

The SCLD-symptom association was established via the Human Symptoms Disease Network (HSDN) [48]. The term frequency (TF) and inverse document frequency (IDF) values from HSDN were used as the association scores to build the network. All the symptoms associated with SCLD were extracted from HSDN patients on the basis of their association scores > 2. In HSDN, obesity is considered a symptom rather than a disease. Therefore, the SCLD disease-symptom network included three disease nodes, namely, asthma, pulmonary hypertension, and pulmonary fibrosis, with obesity as the symptom.

#### Selection of SCLD symptoms through clinical references

Our literature survey revealed that two updated editions of clinical reference books, namely, Harrison’s Principles of Internal Medicine 21st edition and Davidson’s Principles and Practice of Medicine 20th edition, are the most widely used reference books for disease-associated symptom literature among medical practitioners (Loscalzo et al. [43]. Our study used these two reference books to extract various symptoms related to SCLD: asthma, pulmonary hypertension, obesity, and pulmonary fibrosis. In accordance with the literature, we treated obesity as a disease rather than a symptom.

#### Selection of coexisting SCLD symptoms

The HSDN and literature symptoms were merged to represent the coexisting symptoms. These symptom associations with SCLD were further verified via various publicly available human disease-symptom databases, such as Medical Subject Headings (MeSH), the Unified Medical Language System (UMLS), the Human Phenotype Ontology (HPO), and the Mala Card Disease ID (MCID).

### Seasonal linkage of SCLD symptoms

#### GT analysis

We used GT to study the temporal trends in the web search via the monthly relative search volume (RSV) of SCLD symptoms related to obesity as the benchmark [31]. We selected obesity as the benchmark symptom because of its comorbid seasonal linkage with SCLD, such as pulmonary hypertension, pulmonary fibrosis, and asthma [30]. The RSV ranged between 0 and 100, with 100 being the highest search proportion per month. The monthly RSVs for 12 SCLD symptoms were downloaded in *.csv format with and without reference diseases from 2015–2019 across India. In the query, “all categories” and “all types of web search” were used, which are the default settings of GT. We selected “India” using the country settings for the period of Jan 2015–Dec 2019. We selected the country of India for the following reasons: the world's largest population of 1.5 billion with diverse ethnicities, different lifestyles, almost all types of extreme weather and seasonal patterns, more than seventeen official linguistics, and English as one of the major common languages. During the GT search, symptoms were modified to bring their generic meaning closer to clinical meaning. In this context, the following terms were modified: fever, high fever,headache, severe headache; appetite change/weight gain, obesity; cough, dry cough; and sleep difficulty, sleep deprivation.

#### Time series data analysis

The Pandas and NumPy packages of Python version 3.12.1 were used in our entire analysis. Data processing and statistical analysis were carried out via Trend 0.1 of Python [4]. Time series-related seasonal decompositions were carried out via "Statsmodels", which refers to a statistical modeling package in Python that provides a comprehensive library of statistical and econometric tools for data analysis [39]. The seasonal data component decomposition of each search term time series was carried out via the local regression method (LOESS) [35]. Matplotlib was used to plot multiple time series data in the same graph, and the Seaborn library was used to plot a graph with the help of the Matplotlib package [28]. The time series decomposition was used to measure the strength of the trend and seasonality in the time series [44].

## Results

### HSDN-based common symptoms

**Fig. 1:**
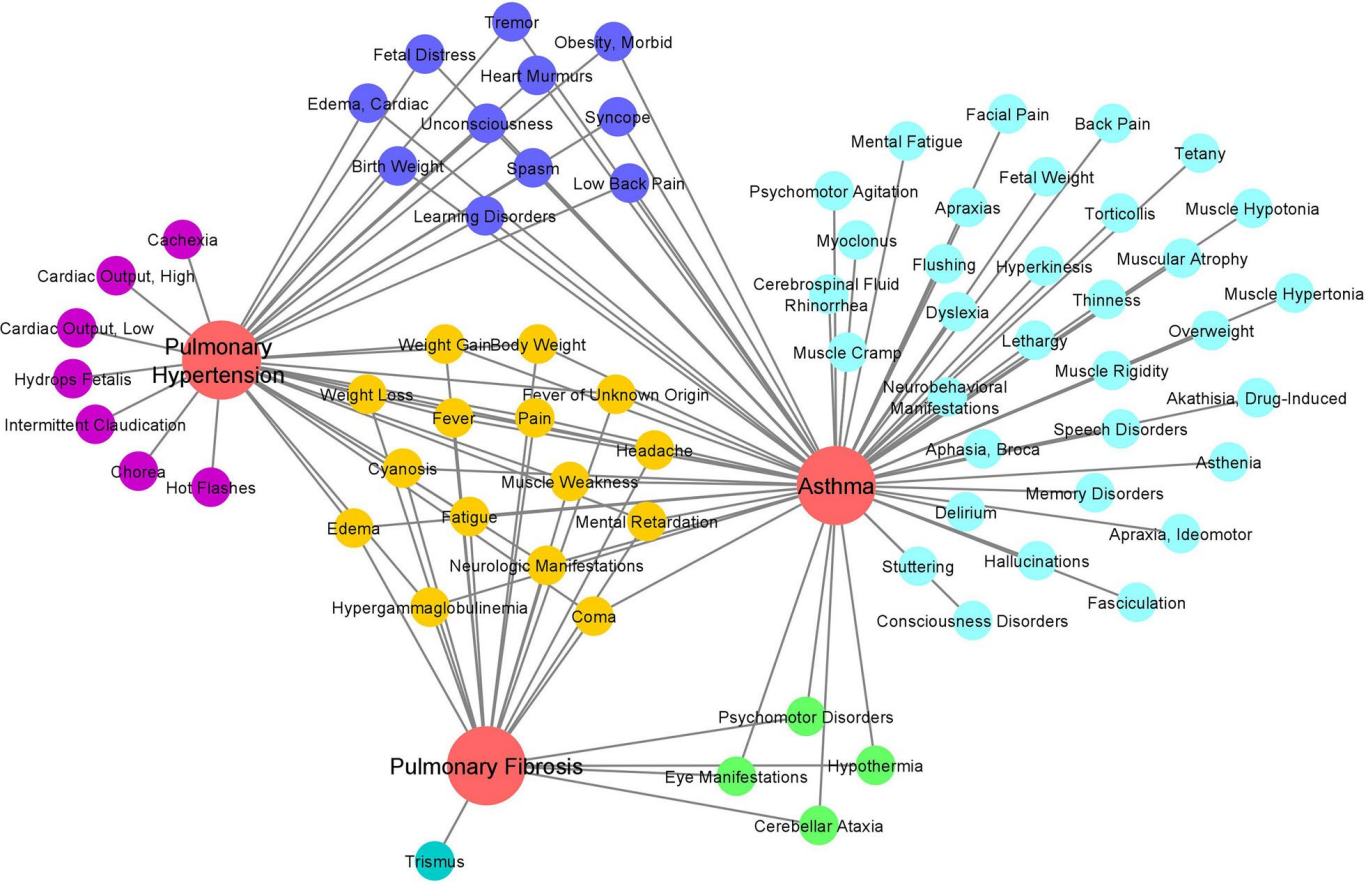
SCLD namely Asthma, Pulmonary Fibrosis, and Pulmonary Hypertension were encircled in red color. The symptoms of each disease extracted from HSDN with TF-IDF scores > 2.0 were shown in different colors. The symptoms shared among all three diseases are shown in yellow color. The symptoms shared between Asthma and Pulmonary fibrosis were shown in green color. The symptoms shared between Asthma and Pulmonary hypertension were shown in indigo color. The symptoms associated with only one disease were represented in cyan color (Asthma), turquoise color (Pulmonary Fibrosis), and magenta (Pulmonary Hypertension)

As mentioned earlier, considering obesity as a symptom, a comprehensive list of symptoms associated with SCLD, namely, pulmonary hypertension, pulmonary fibrosis, and asthma, was extracted from HSDN. We explored the associations among various symptoms and diseases via the TF-IDF weighted values from HSDN. This network filtered out 71 symptoms strongly associated with SCLD. We observed that almost half of the symptoms in the network were linked to asthma, followed by pulmonary hypertension and pulmonary fibrosis (Fig. 1).

**Fig. 2:**
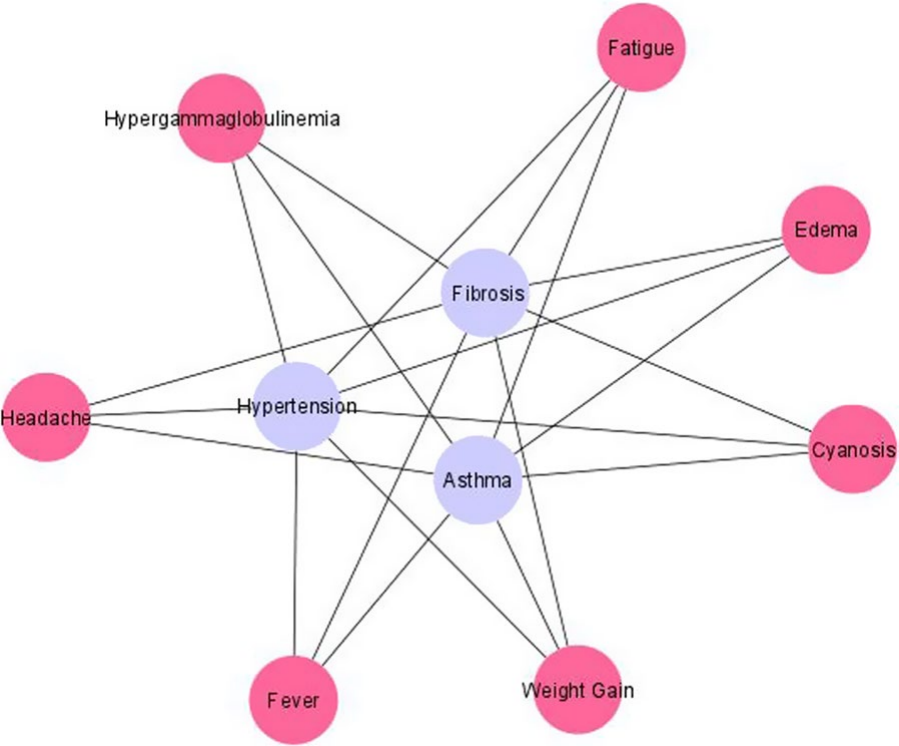
The sub-network of common symptoms of SCLD with TF-IDF score > 2.0 were shown. The disease nodes were represented in purple, and 7 common symptom nodes were shown in pink, encrypted with the corresponding symptom names

To further assess the close relationship, symptom nodes commonly shared among the three disease nodes were dissected. In search of closely associated symptoms, the TF-IDF values were limited to > 2.0. The resulting subnetwork displayed seven common symptom nodes shared among the three disease nodes (Fig. 2). The common symptoms cyanosis, hypergammaglobulinemia, and weight gain were strongly associated with all the disease nodes (Table 1). Although obesity is not associated with HSDN, the corresponding weight gain symptoms are common symptoms with high association scores.

**Table 1:** TF-IDF score from HSDN based on seven common symptoms of SCLD

Common symptoms	TF-IDF Scores		
	Pulmonary hypertension	Asthma	Pulmonary fibrosis
Fever	3.09	26.61	9.91
Weight gain (*)	8.92	14.86	7.43
Cyanosis (*)	67.99	20.92	27.89
Edema	12.51	31.58	2.38
Fatigue	7.96	29.58	3.41
Hypergammaglobulinemia (*)	8.39	30.23	26.86
Headache	6.192	43.34	2.65

The symptoms with high scores in all three diseases were highlighted (*)

### Literature-based common symptoms

**Fig. 3:**
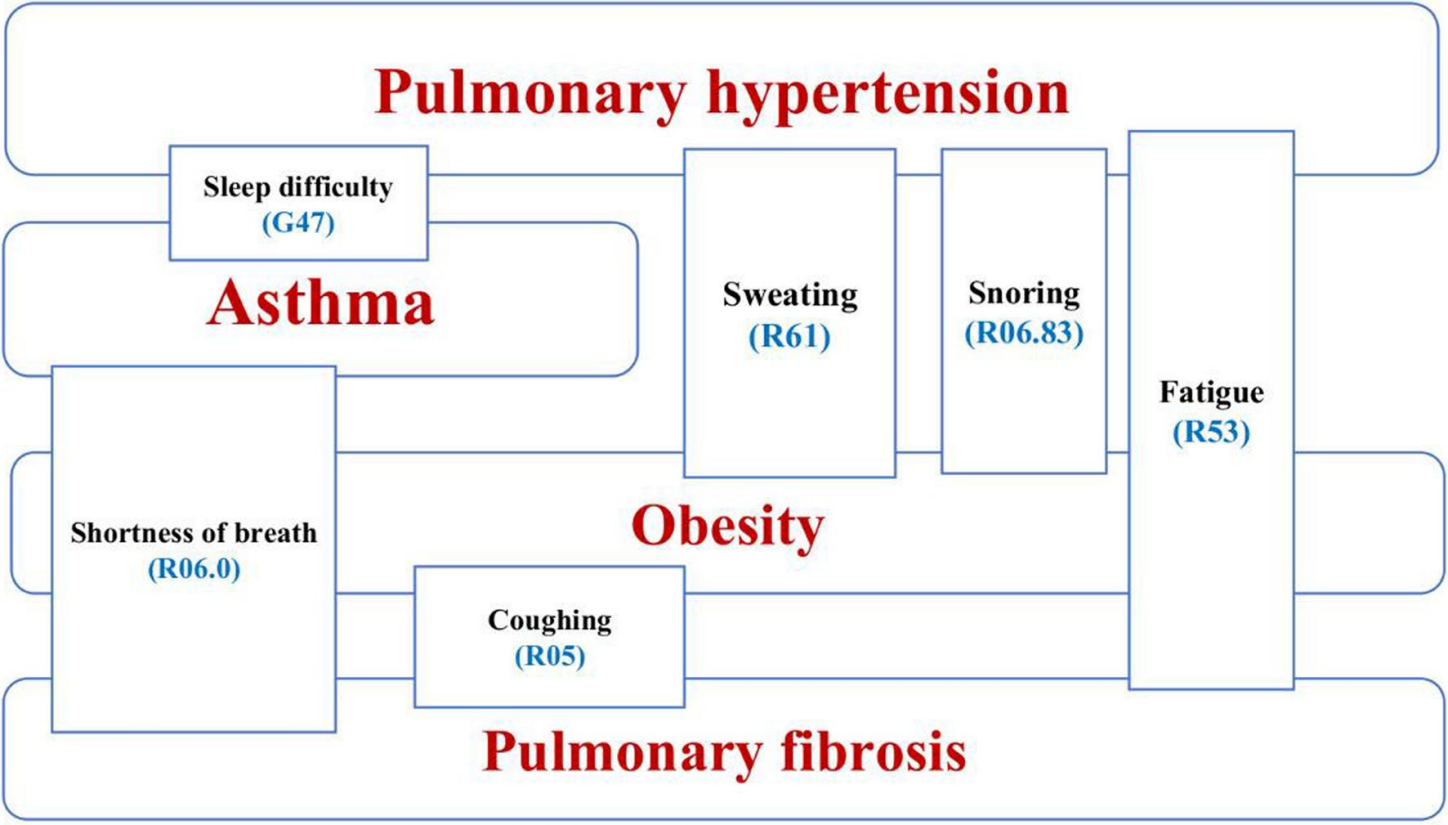
The Venn diagram of SCLD symptoms collected from literature resources was shown. The diseases were depicted in red color and ICD10 of symptoms were in blue. Note that the symptoms namely fatigue and shortness of breath were shared among three diseases, and the remaining 5 symptoms were shared among two diseases

The collection of generic clinical SCLD symptoms from the two clinical literature sources revealed 35 symptoms (Table 2). Importantly, these two studies considered obesity a disease [19, 26, 40]. The Venn diagram revealed that two or more diseases were associated with six symptoms. These six symptoms represent a set of common symptoms in the literature (Fig. 3).

**Table 2:** List SCLD symptoms collected from medical literature resources

S.No	SCLD	Symptoms from literature resources	ICD-10 Codes
1	Hypertension	Headache	R51
		Chest pain	R07.89
		Palpitations	R00.2
		Irregular heart beats	R00
		Nosebleeds (Epistaxis)	R04.0
		Fainting spells (syncope)	R55
		Muscle tremors	M62.00
		Vision changes	H53.2
		Fatigue	R53
		Sleep disturbance	G47
		Sweating	R61
		Snoring	R06.83
2	Asthma	Wheezing or whistling sound during breathing	R06.2
		Sleep difficulties	G47.9
		Chest tightness	R07.89
		Increased mucus production	R09.3
		Breathlessness (Dyspnoea)	R06.0
		Coughing	R05
		Sleep disturbance	G47
3	Pulmonary fibrosis	Hemoptysis	R04.2
		Loss of appetite	F50
		weight loss	R63.4
		Shallow and fast breathing	R06.89
		Clubbing finger	R68.3
		Breathlessness (Dyspnea)	R06.0
		Coughing	R05
		Fatigue	R53
4	Obesity	Pot belly	E66.01
		Binge eating	F50.8
		Inability to do sudden physical activity	Y93
		Breathlessness (Dyspnea)	R06
		Fatigue	R53
		Sweating	R61
		Snoring	R06.83

### SCLD coexisting symptoms

We combined the coexisting symptoms from HSDN and the literature to represent a panel of the most promising coexisting symptoms of SCLD. In this panel, one symptom, namely, weight gain/appetite change, was common. As mentioned in the methodology, weight gain/appetite change was used instead of obesity. Overall, we obtained 12 common symptoms representing coexisting symptoms from the two different sources, HSDN and the literature (Table 3). These 12 coexisting symptoms were further verified via the various disease-symptom databases listed in the methodology (Supplementary Table S1).

**Table 3:** List of common symptoms of SCLD from HSDN and literature

SCLD common symptoms		SCLD Co-existing symptoms
HSDN common symptoms	Literature common symptoms	Combined common symptoms
Cyanosis	Shortness of breath	Cyanosis
Hypergammaglobulinemia	Sweating	Hypergammaglobulinemia
Weight gain (Obesity)	Snoring	Weight gain/Appetite change (Obesity)
Fatigue	Sleep deprivation	Fatigue
Headache	Appetite change (Obesity)	Headache
Edema	Coughing	Edema
Fever		Fever
		Shortness of breath
		Sweating
		Snoring
		Sleep deprivation
		Coughing

These common symptoms were combined to represent the “12 co-existing symptoms of SCLD”

### GT analysis in India

**Fig. 4:**
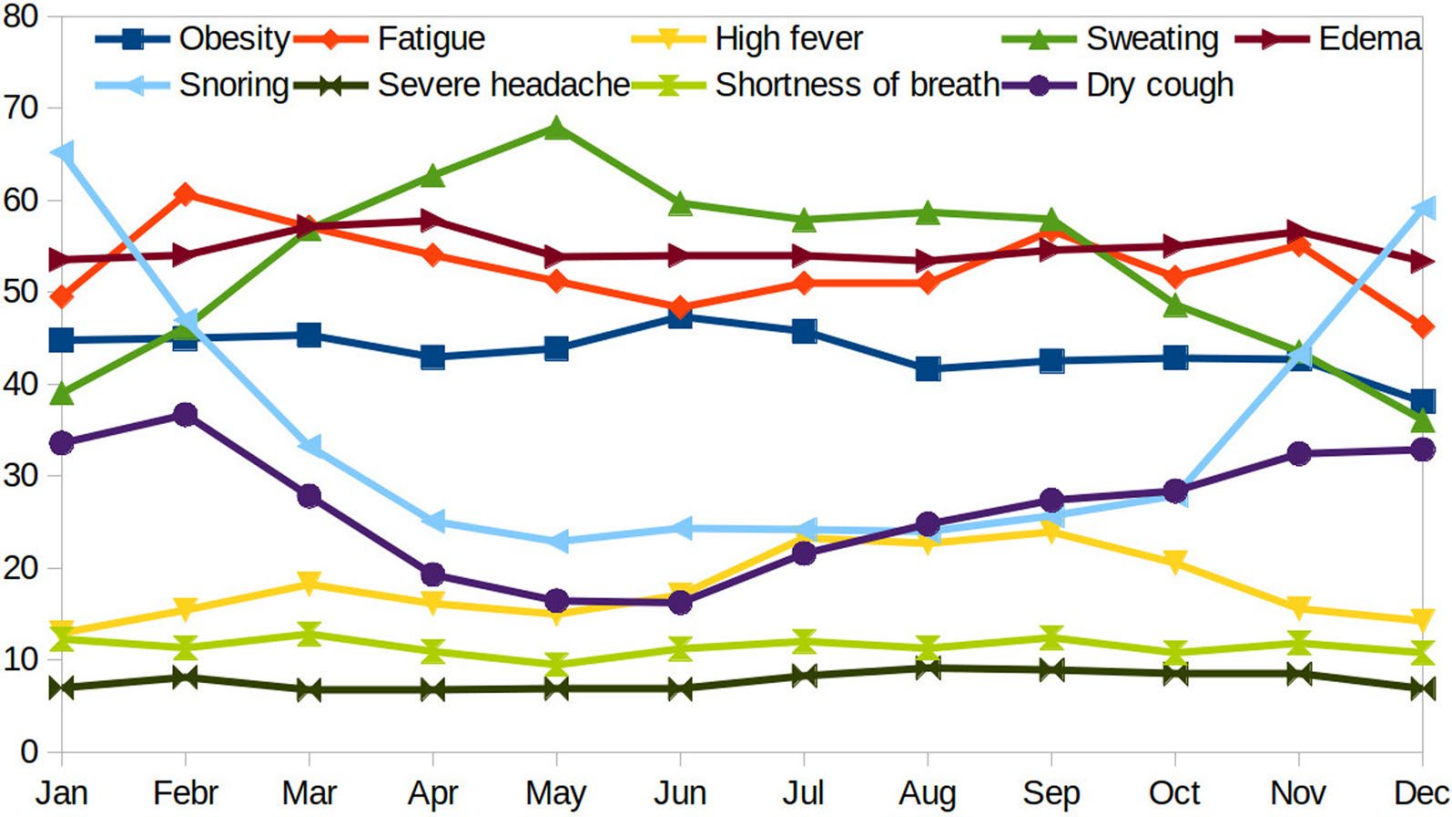
The Indian monthly average RSV of 9 co-existing symptoms of SCLD for the Years 2004 to 2009 were shown in different colors. Note that out of 9 symptoms with RSV, the four symptoms namely fatigue, obesity, edema, and sweating showed their average RSV > 43 with obesity as the benchmark

Using obesity as the benchmark keyword, the monthly varying RSVs for the 12 coexisting symptoms were analyzed for the period 2015–2019 in India. We categorized the low RSV symptoms on the basis of their minimum average RSV of a year, which was less than 10, in comparison with a maximum RSV of 100. Among the twelve symptoms, nine symptoms presented an RSV > 10, and three symptoms, namely, sleep deprivation, hypergammaglobulinemia, and cyanosis, presented an RSV < 10, with obesity as the benchmark (Supplementary Table S2A). For this reason, we further collected RSVs of these three symptoms without obesity as the benchmark (Supplementary Table S2B). Even in this case, the symptoms of hypergammaglobulinemia yielded low RSV values, and the data were unavailable for several months. The average RSV of obesity in India for the entire 5-year period was 43.53. Among the nine symptoms associated with RSV, four symptoms, namely, fatigue, obesity, edema, and sweating, presented average RSVs > 43, with obesity as the benchmark. The prevalence of these symptoms in India is almost twice that of the other five symptoms, namely, high fever, shortness of breath, snoring, dry cough, and severe headache (Fig. 4).

### Seasonality in SCLD symptoms

**Fig. 5:**
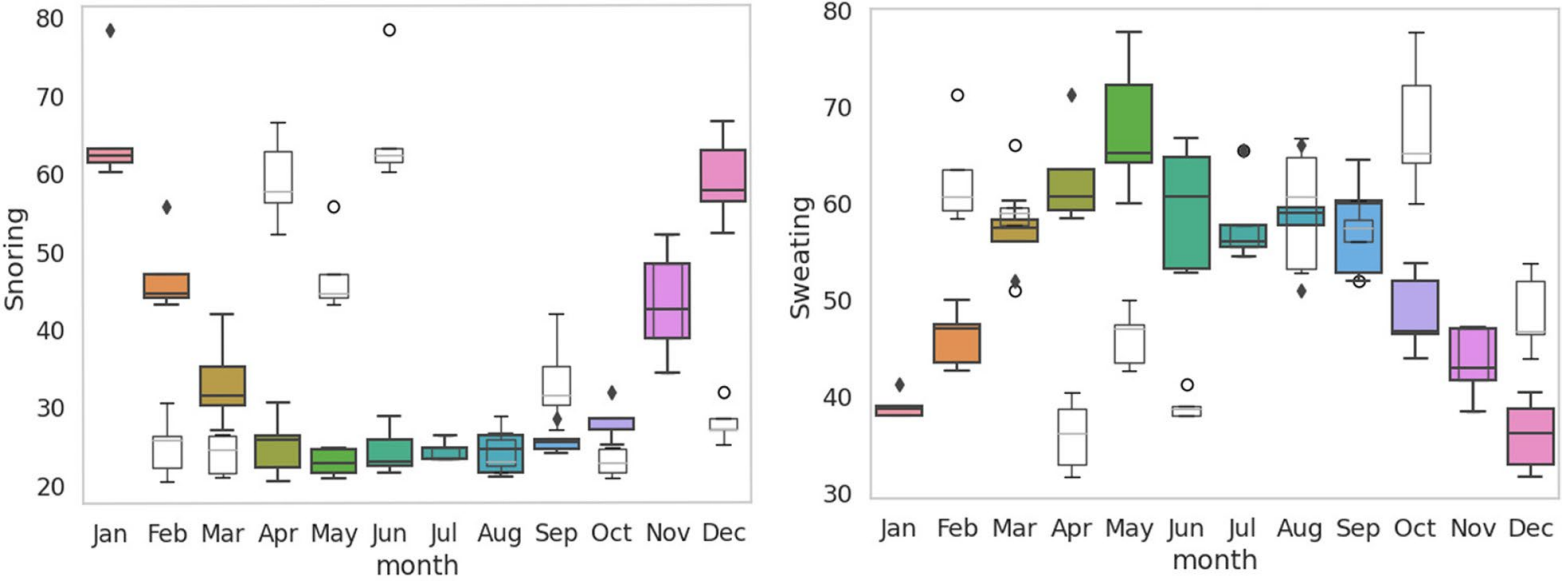
Box plot of seasonal average levels of sweating and snoring is shown. The horizontal solid lines indicate the mean values and range whereas the top and bottom of the box indicate the 75th and 25th percentiles. These two symptoms defined two seasons summer and winter with equal 6-month lengths that spanned a single calendar year such as two semesters (e.g. December–July vs. July–December). Both symptoms showed almost opposite minimum and maximum values in the entire year. Note the apparent prominent changes in the RSV during November and March peaks before the RSV could reach either minimum or maximum values

**Fig. 6:**
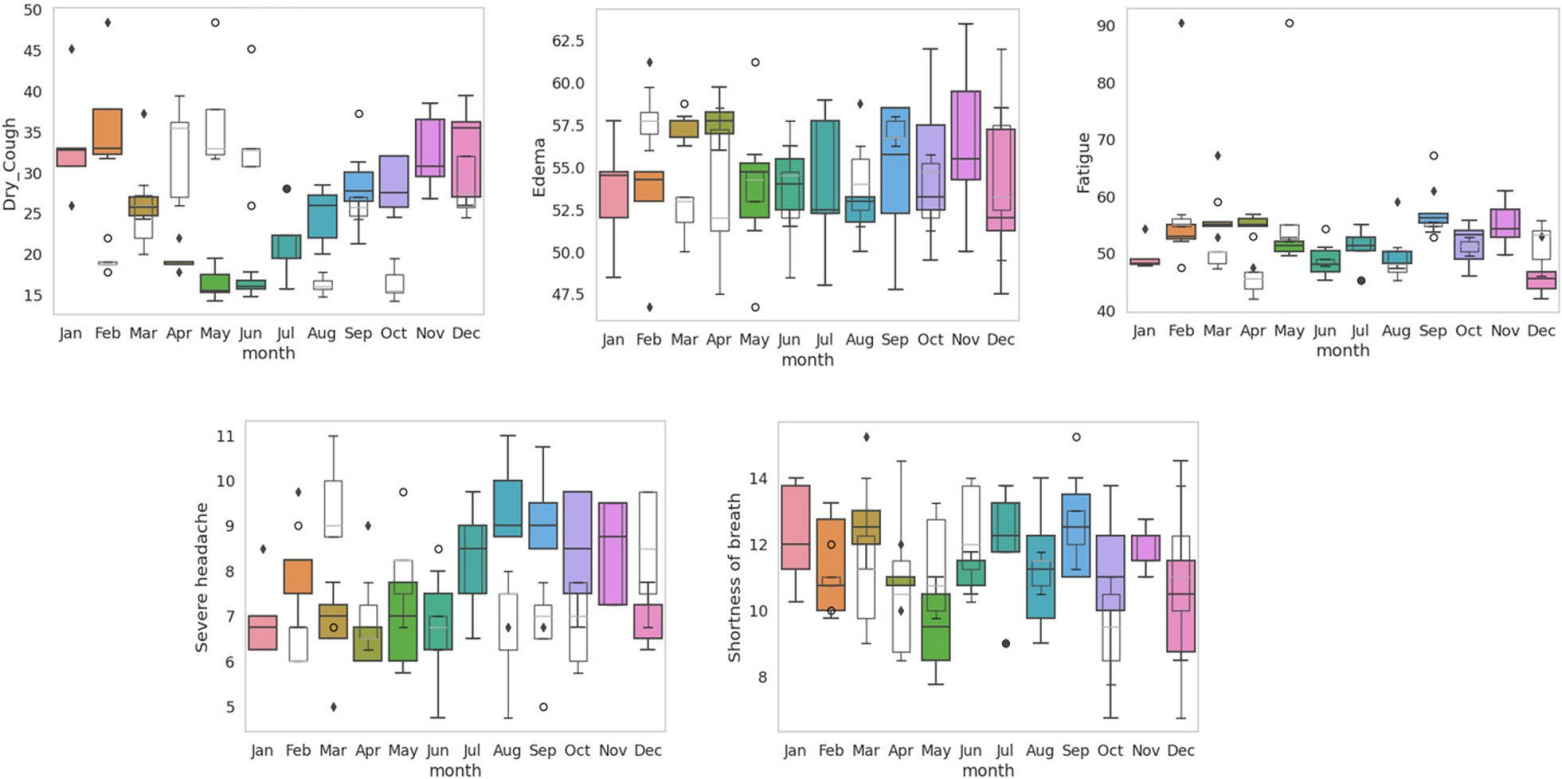
Box plots of seasonal average levels of severe headache, fatigue, dry cough, shortness of breath, and edema are shown. All these five symptoms are well defined in four Indian seasons spanned in a single calendar year (a) winter (December to February), summer (March to May), monsoon or rainy season (June to September), and a post-monsoon period (October and November). Note the apparent prominent changes in the RSV during November and March peaks before the RSV could reach either minimum or maximum values

**Fig. 7:**
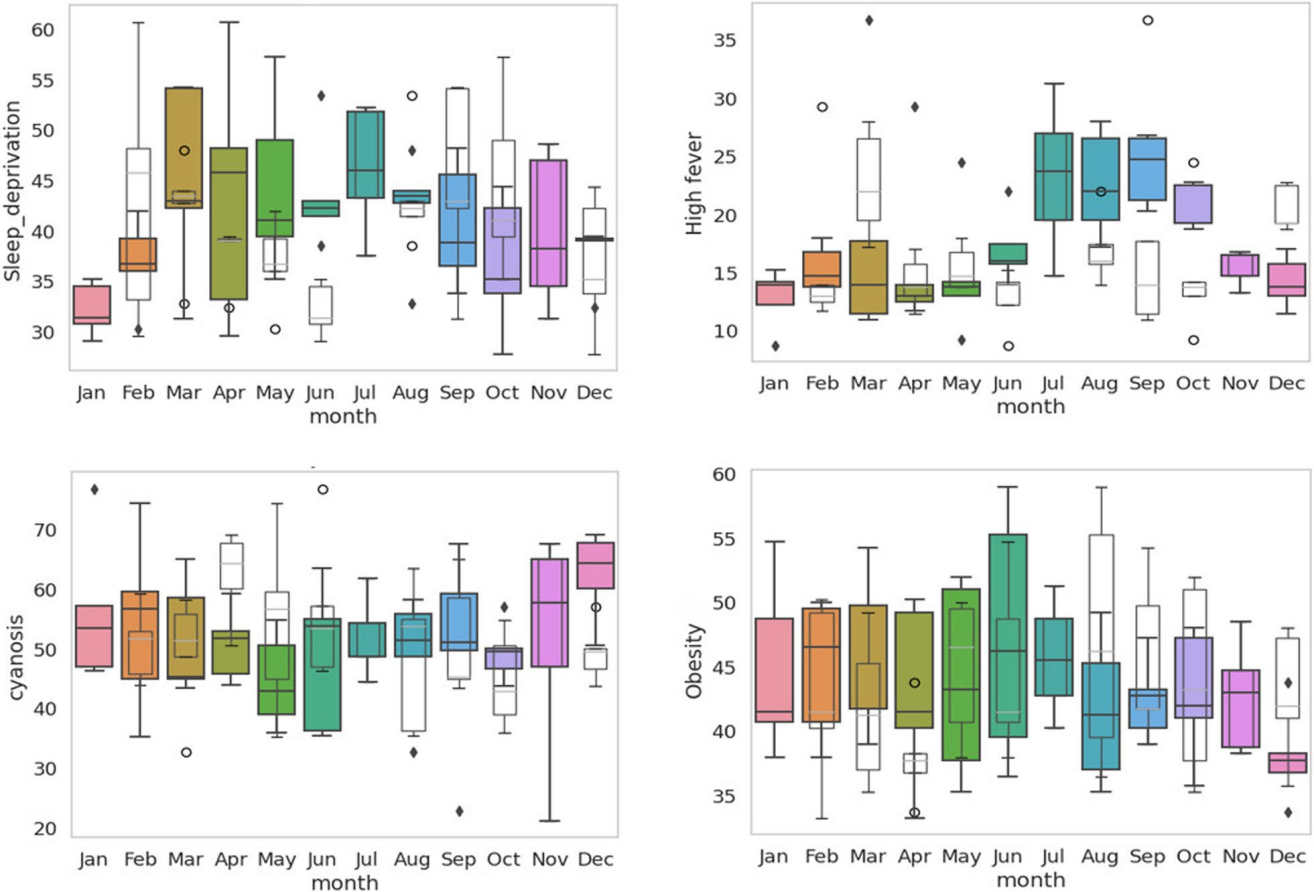
Box plots of seasonal average levels of high fever, obesity, cyanosis, and sleep deprivation are shown. All these four symptoms moderately defined four Indian seasons spanned in a single calendar year (a) winter (December to February), summer (March to May), monsoon or rainy season (June to September), and a post-monsoon period (October and November). Note the apparent changes in RSV during November and March peaks before the RSV could reach either minimum or maximum values

Seasonality analysis was carried out via boxplot analysis of the monthly average RSV of each symptom for the five years. Two symptoms, sweating and snoring, showed two seasonal peaks (Fig. 5). The five symptoms, namely, dry cough, edema, fatigue, shortness of breath, and severe headache, showed four strong seasonal peaks at 3-month intervals (Fig. 6). The four symptoms, namely, sleep deprivation, high fever, obesity and cyanosis, presented four moderate seasonal peaks at 3-month intervals (Fig. 7). These four-month quarters differ from the four seasonal quarters of India, such as winter (December to February), summer (March to May), the monsoon or rainy season (June to September), and the postmonsoon period (October and November). Overall, we noticed an apparent prominent change in the RSV during the November and March seasonal peaks before they reached either minimum or maximum values.

### Seasonal trends in SCLD symptoms

**Fig. 8:**
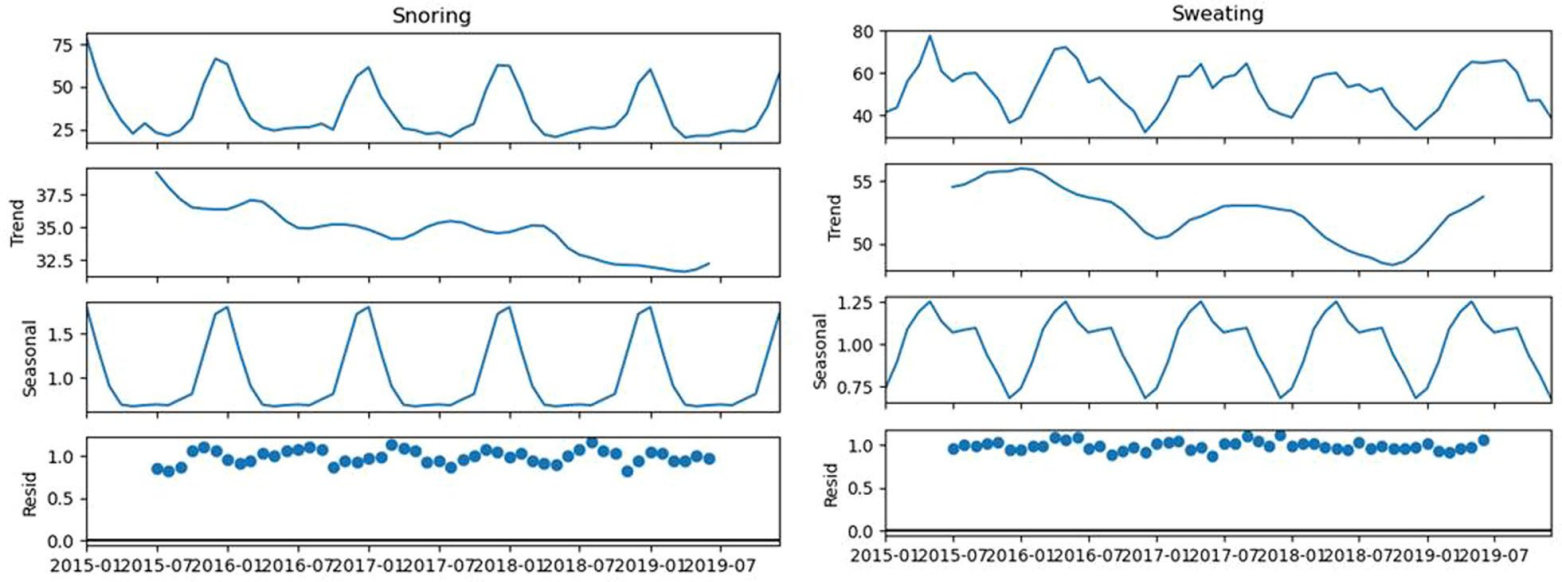
The seasonal decomposition of seasonal symptoms of sweating and snoring for the monthly averaged RSV from 2015 to 2019 with obesity as the benchmark symptom. Note the opposite seasonal trends in the sweating and snoring

**Fig. 9:**
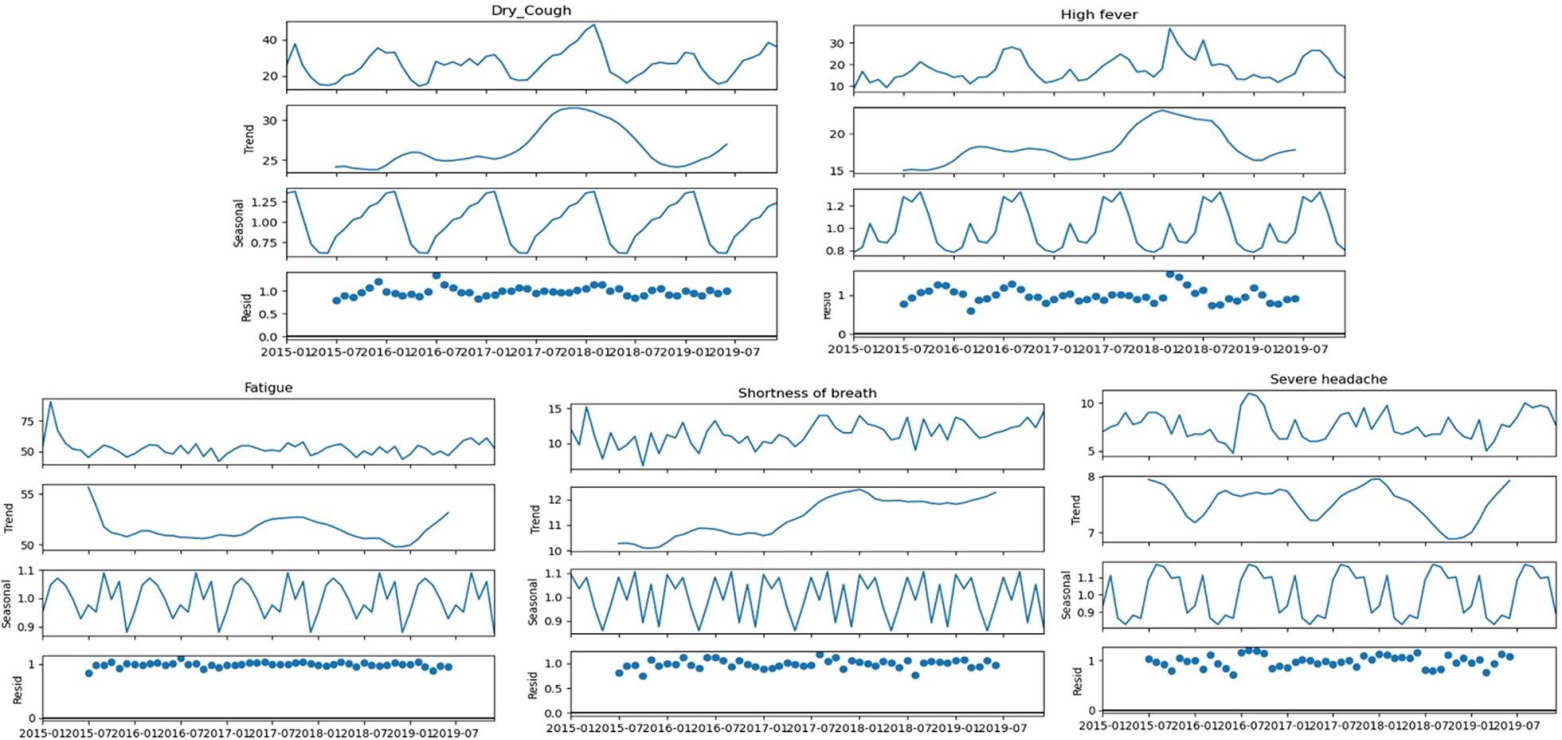
The seasonal decomposition of quarterly seasonal symptoms of dry cough, high fever, fatigue, shortness of breath, and severe headache for the monthly averaged RSV from 2015 to 2019 with obesity as the benchmark symptom. Note the repeating distinct seasonal patterns in all the symptoms

**Fig. 10:**
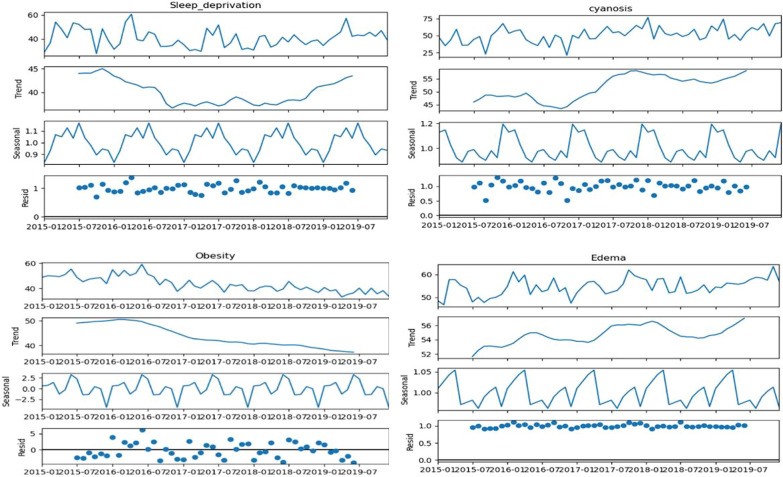
The seasonal decomposition of quarterly seasonal symptoms of cyanosis and sleep deprivation for the monthly averaged RSV from 2015 to 2019 without obesity as the benchmark symptom. The seasonal decomposition of quarterly seasonal symptoms of obesity and edema with the benchmark is also shown. Note the repeating distinct seasonal patterns in all the symptoms

Using obesity as the benchmark keyword, the monthly varying RSVs for the 9 symptoms (including obesity) were analyzed for seasonal trends from 2015 to 2019. The seasonal decomposition analysis was carried out on the 9 symptoms. Similarly, for the remaining two symptoms, namely, cyanosis and sleep deprivation, seasonal decomposition analysis was carried out on the RSV without the obesity benchmark. On the basis of their overall seasonal strength value, we classified symptoms into strong (Fs > 0.9), moderate (Fs > 0.4), and weak (Fs < 0.4) symptoms. Two symptoms, namely, sweating (0.906) and snoring (0.957), with biseasonal linkages presented strong seasonal components (Fig. 8). The 5 symptoms with quarterly seasonal linkages, namely, dry cough (0.851), high fever (0.445), fatigue (0.639), severe headache (0.501) and shortness of breath (0.490), presented moderate seasonal components (Fig. 9). The remaining 4 symptoms with quarterly seasonal linkages, namely, sleep deprivation (0.319), cyanosis (0.272), dementia (0.268), and obesity (0.391), presented weak seasonal components (Fig. 10).

## Discussion

In the present study, we developed an approach to establish a panel of major seasonally sensitive symptoms associated with SCLD to screen the so-called “seasonally sensitive population” from the normal population. Using HSDN and literature resources, we could narrow down to 12 coexisting major symptoms associated with SCLD. Despite the known limitations of GT, our earlier studies well demonstrated the global seasonal patterns in SCLD. On the basis of this evidence, SCLD is seasonally comorbid, and we hypothesize that their associated coexisting symptoms are also expected to have a seasonal relationship. To address this issue clinically, the study of the seasonal linkage of symptoms and electronic health records of larger populations for a minimum tracking period of one year are needed. To our knowledge, no such clinical records are available for a larger population to validate our hypothesis. To meet this requirement, we exploit GT datasets from India for the last 5 years (2015–2019) before the start of the COVID-19 pandemic. As expected, our results revealed that 9 out of 12 coexisting symptoms of SCLD were seasonally linked. With obesity as the benchmark, due to low RSV, it is challenging to establish comorbid seasonal changes in cyanosis, sleep deprivation/disorder/difficulties, and hypergammaglobulinemia. However, enough evidence worldwide suggests that sleep deprivation/sleep difficulty and cyanosis are significantly affected by seasonal changes (Cizza et al.) [24, 41] [3]. Significant seasonal changes in IgA levels have also been observed in clinical studies, strongly suggesting the association of hypergammaglobulinemia with seasons [42] [45]. Moreover, without a benchmark, sleep deprivation and cyanosis symptoms indicated the presence of mild to moderate seasonal components in GT. Our study's overall seasonal component analysis supported our hypothesis that all 12 significant SCLD symptoms are seasonally associated or seasonally comorbid in the Indian population. Further GT studies and clinical support for the worldwide population are necessary to make the hypothesis more generic and valid.

There are several well-documented clinical cases where these 12 symptoms coexist or co-occur, with strong seasonal associations in the winter, except for sweating and sleep deprivation/difficulty. In such cases, the incidence and severity of shortness of breath symptoms increase during the winter season [46]. Patients with prolonged and persistent shortness of breath commonly present with several comorbid seasonal symptoms, such as swelling in the ankles and feet (edema), unintentional weight loss with loss of appetite (obesity), unusual fatigue, sweating, fever, chronic cough, blue discoloration of the lips or fingertips (cyanosis), and light-headedness (headache) [6]. Second, individuals subjected to severe sleep disorders in winter were found to have the following comorbid seasonal symptoms: breathing disorders, snoring, coughing, and shortness of breath [8]. Third, obese individuals tend to develop hypoventilation syndrome with an increase in the severity of the following comorbid seasonal symptoms: sleep disorders, fatigue, headache, and shortness of breath. Fourth, owing to their improper ventilatory response, unacclimatized individuals at high altitudes also have increased severity of seasonal symptoms such as headache, fatigue, sleep deprivation, and loss of appetite (obesity) [17, 21]. Overall, it is reasonable to summarize that individuals with improper ventilatory response during a seasonal change are most likely to develop severe symptoms of one or more of the 12 coexisting symptoms. On this basis, we classified these 12 coexisting seasonal symptoms of SCLD as “seasonally sensitive comorbid symptoms (SSC symptoms)”, and further clinical studies are necessary to verify our claim. Furthermore, several respiratory disorders associated with improper ventilatory response, such as COPD, asthma, pulmonary fibrosis, pulmonary hypertension, and tuberculosis, are also seasonally linked [9, 11, 14]. Evidently, most of the SSC symptoms commonly shared among the above diseases further suggest that the wide range of clinical implications of SSC symptoms should be explored further [13, 16, 22].

Our studies revealed that the seasonality patterns of most of the coexisting symptoms were consistent with Indian seasonal changes. In this analysis, we observed six months of seasonal components related to snoring and sweating. However, the remaining symptoms presented four mild to moderate seasonal variations. Studies carried out in various geological locations worldwide also reported seasonality in these coexisting symptoms of SCLD. Interestingly, our analysis indicated that these coexisting symptoms experienced significant changes during March and November. Only limited clinical studies have been conducted in India to study the seasonal patterns in these coexisting symptoms. Indirectly, it is intuitive to propose that the seasonal change effect on the seasonally sensitive population is periodic with Indian seasons. Our study strongly necessitates further clinical studies on the effects of seasonal changes on these 12 symptoms to address the seasonally sensitive Indian population.

Several studies have provided enough evidence that GT can be used to monitor several disorders in real-time indirectly [37]. Individual disease-based GT monitoring of the health status of the human population is more laborious, complex, expensive, and less effective than monitoring symptoms. To our knowledge, no alternate human population health monitoring platform or tool is available to monitor their general health status in real-time for the entire city, region, state, or country. In this context, SSC symptoms have several implications if integrated with GT. First, these SSC symptoms are not only part of seasonal disorders but also major coexisting symptoms in the plethora of lifestyle disorders, neurological disorders, communicable disorders, and non-communicable disorders (Supplementary Table S3). Second, these 12 SSC symptoms could be easily monitored through RSV scores in real time 24 × 7 via a mobile application and internet for the entire country or a specific region. Moreover, their outcomes are readily correlated with various healthcare stakeholders for verification. Third, we further classified the SSC symptoms into strong (Class A), moderate (Class B), and weak (Class C) symptoms based on their Indian RSV scores and seasonal components. The Indian Class A seasonal symptoms are sweating and snoring. The Class B Indian symptoms are severe headache, fatigue, dry cough, shortness of breath, and high fever. The Class C Indian symptoms are obesity, edema, high fever, sleep deprivation, hypergammaglobulinemia, and cyanosis. The subset of symptoms in each class is subject to variation depending upon the RSV scores of the geographical location, time, and reference symptoms. However, studying this country-wide classification of SSC symptoms may serve as a standard reference for comparing regional or time-specific variations in each subset of symptoms. For example, the Indian capital, Delhi, showed a completely different shuffling of symptoms in each class until October 2023 (Supplementary Table S4). Significantly, in October 2023, in Delhi, the symptoms of sleep deprivation shifted from Class C to A. This shift could be readily correlated with the current drastic drop in the Delhi air quality index, which is well below the normal level [12]. With several limitations listed below, SSC symptoms could be used to develop a promising handy tool in tune with GT to indirectly monitor the general public health status of the entire globe, country, region, and city in real-time.

In our study, we enlisted 12 SSC symptoms without any clinical studies to support the minimum number of common symptoms required to monitor the common health status of the seasonally sensitive population. However, these 12 symptoms are the starting point for developing the minimum number of common symptoms required to monitor seasonally sensitive populations. We used GT to indirectly support the seasonal association of 12 SSC symptoms and their seasonal comorbid nature. Moreover, many SSC symptoms are modified to increase their medical relevance during their GT search. Although Google supports 19 Indian languages, we limited our search query to English. Therefore, our GT result does not completely reflect the Indian public search interest in a particular health symptom; it simply reflects the relative search trend in a particular topic. Therefore, careful interpretation is needed to correlate the outcomes of GT with those of clinical studies. In addition, several review articles have well-documented the general limitations and drawbacks. [2, 25]. We warrant that the user be aware of these GT limitations before correlating their clinical outcome with SSC symptoms.

## Supplementary Information

Additional file 1.

Additional file 2.

Additional file 3.

Additional file 4.

Additional file 5.

## Data Availability

The details of data availability were given below: SCLD symptoms extracted from various Disease Databases such as Medical Subject Headings (MeSH), Unified Medical Language System (UMLS), Human Phenotype Ontology (HPO), and Mala Card Disease ID (MCID). were given in the supplementary Table S1. Google Trends (https://trends.google.com/trends/) was used to extracted residual search volumes were given in the supplementery tables S2A, S2B and S4. The supporting evidence for SCLD symptoms association with various diseases extracted form Pubmed were given in S3 (https://pubmed.ncbi.nlm.nih.gov/).

## Abbreviations

SCLD: Season-sensitive comorbid lifestyle diseases
GT: Google trends
HSDN: Human symptom disease network
SSC: Seasonal sensitive comorbidity
RSV: Relative search volume
