# Supplementary material for: Google trend analysis of the Indian population reveals a panel of seasonally sensitive comorbid symptoms with implications for monitoring the seasonally sensitive human population
Source: Popul Health Metr. 2024 Dec 30;22:40. doi: 10.1186/s12963-024-00349-7 (PMC11686857; doi:10.1186/s12963-024-00349-7)
Supplement: Supplementary file 1 — Additional file 1. [file 12963_2024_349_MOESM1_ESM.docx]

Supplementary Table S1. SCLD symptoms were obtained from various Disease Databases

| **Disease** | **Database** | **ICD-10 Codes** |
| --- | --- | --- |
| Hypertension (I10) | MeSH (C1840376) | High systemic arterial blood pressure (I10) |
|  | UMLS CUI(COO6973) | Abnormal increase in the average blood pressure (I10) |
|  | HPO (HP:0004972) | Abnormal increase in the average blood pressure (I10) |
|  | MCID (HYP595) | Abnormally high blood pressure in the arteries (I10) |
| Asthma (J45) | MeSH (DOO1249) | Wheezing (R06.2)  Dyspnea (R06.02) |
|  | UMLS CUI (C0004096) | Wheezing (R06.2)  Dyspnea (R06.02) |
|  | HPO (HP:0002099) | Coughing (R05)  Wheezing (R06.2)  Dyspnea (R06.02) |
|  | MCID (AST005) | Chest tightness (R07.89)  Shortness of breath (R06.02)  Mucus production (J45.20)  Coughing (R05)  Wheezing (R06.2) |
| Obesity (E66.9) | MeSH (D009765) | High body weight (E66) |
|  | UMLS CUI (C0028754) | Overeating (E66.0)  Eating high-fat foods (F50.8)  Not being physically active (Y93) |
|  | HPO (HP:0001513) | Accumulation of excess fats (E66) |
|  | MCID (BDY004) | Decreased resting energy expenditure (Y93) |
| Pulmonary fibrosis (J48.10) | MeSH (D011658) | Progressive Dyspnea (R06.02) |
|  | UMLS CUI (C0034069) | Shortness of breath (R06)  Dry cough (R05)  Fatigue (R53)  Weight loss (R63.4)  Aching muscles and joints (M25.50)  Clubbing of fingers (R68.3) |
|  | HPO (HP:0002206) | Irreversible impairment of alveolar oxygen transfer (J69.2) |
|  | MCID (PLM036) | Progressive shortness of breath (R06)  Haemoptysis (R04.2)  snoring (R06.83) |
