# Supplementary material for: Google trend analysis of the Indian population reveals a panel of seasonally sensitive comorbid symptoms with implications for monitoring the seasonally sensitive human population
Source: Popul Health Metr. 2024 Dec 30;22:40. doi: 10.1186/s12963-024-00349-7 (PMC11686857; doi:10.1186/s12963-024-00349-7)
Supplement: Supplementary file 2 — Additional file 2. [file 12963_2024_349_MOESM2_ESM.doc]

Supplementary Table S2A. Relative Search Volume (RSV) of SCLD symptoms with the benchmark in India for the period from Jan 2015 to 2019

| **Month-Year** | **Obesity** | **Fatigue** | **High fever** | **Sweating** | **Edema** | **Snoring** | **Severe headache** | **Shortness of breath** | **Dry cough** |
| --- | --- | --- | --- | --- | --- | --- | --- | --- | --- |
| Jan 2015 | 48.75 | 54.25 | 8.75 | 41.25 | 48.5 | 78.5 | 7 | 12 | 26 |
| Feb 2015 | 50 | 90.5 | 16.75 | 43.5 | 46.75 | 55.75 | 7.5 | 9.75 | 37.75 |
| Mar 2015 | 49.75 | 67.25 | 11.5 | 56 | 57.75 | 42 | 7.75 | 15.25 | 25.75 |
| Apr 2015 | 49.25 | 56.75 | 13 | 63.5 | 57.75 | 30.5 | 9 | 11 | 19 |
| May 2015 | 51 | 52 | 9.25 | 77.75 | 55.25 | 22.75 | 7.75 | 7.75 | 15.25 |
| Jun 2015 | 55.25 | 51 | 14 | 60.75 | 54 | 28.75 | 8 | 11.5 | 14.75 |
| Jul 2015 | 48.75 | 45.25 | 14.75 | 56 | 48 | 23.25 | 9 | 9 | 15.75 |
| Aug 2015 | 45.25 | 50.25 | 17.25 | 59.5 | 50 | 21.5 | 9 | 9.75 | 20 |
| Sep 2015 | 47.25 | 55.25 | 21.25 | 60 | 47.75 | 24.5 | 8.5 | 11 | 21.25 |
| Oct 2015 | 48 | 53.25 | 18.75 | 53.75 | 49.5 | 31.75 | 6.75 | 6.75 | 24.5 |
| Nov 2015 | 48.5 | 49.75 | 16.75 | 47.25 | 50 | 52 | 8.75 | 11.5 | 30.75 |
| Dec 2015 | 43.75 | 45.5 | 15.75 | 36.25 | 51.25 | 66.5 | 6.5 | 8.5 | 35.5 |
| Jan 2016 | 54.75 | 48.25 | 14 | 39 | 54.75 | 63.25 | 6.75 | 11.25 | 32.75 |
| Feb 2016 | 49.5 | 52.5 | 14.75 | 50 | 61.25 | 44 | 6.75 | 10.75 | 33 |
| Mar 2016 | 54.25 | 55.5 | 11 | 60.25 | 56.75 | 31.5 | 7.25 | 13 | 24.75 |
| Apr 2016 | 50.25 | 55 | 14 | 71.25 | 59.75 | 26.25 | 6 | 10 | 17.75 |
| May 2016 | 52 | 49.5 | 14.25 | 72.25 | 51.25 | 24.5 | 5.75 | 8.5 | 14.25 |
| Jun 2016 | 59 | 48 | 17.5 | 66.75 | 55.5 | 25.75 | 4.75 | 11.75 | 15.75 |
| Jul 2016 | 51.25 | 55 | 27 | 55.5 | 52.5 | 26.25 | 9.75 | 13.25 | 28 |
| Aug 2016 | 49.25 | 48.25 | 28 | 57.75 | 53.25 | 26.5 | 11 | 11.25 | 26 |
| Sep 2016 | 42.75 | 56.25 | 26.75 | 52 | 58.5 | 28.5 | 10.75 | 11 | 27.75 |
| Oct 2016 | 47.25 | 46 | 19.25 | 46.5 | 52.5 | 25 | 9.75 | 10 | 25.75 |
| Nov 2016 | 44.75 | 52.75 | 14.75 | 41.75 | 54.25 | 42.5 | 7.25 | 11 | 29.5 |
| Dec 2016 | 37.75 | 42 | 11.5 | 31.75 | 47.5 | 56.25 | 6.25 | 8.75 | 26 |
| Jan 2017 | 41.5 | 48 | 12.25 | 38 | 52 | 61.5 | 6.25 | 10.25 | 30.75 |
| Feb 2017 | 46.5 | 52 | 13.75 | 47 | 54.75 | 44.5 | 8.25 | 10 | 31.75 |
| Mar 2017 | 41.75 | 54.75 | 17.75 | 58.25 | 56.75 | 35.25 | 6.5 | 11.25 | 27 |
| Apr 2017 | 40.25 | 54.75 | 12.5 | 58.5 | 57 | 25.75 | 6 | 10.75 | 18.75 |
| May 2017 | 43.25 | 52.75 | 13 | 64.25 | 54.75 | 24.75 | 6 | 9.5 | 17.5 |
| Jun 2017 | 46.25 | 50.5 | 16 | 52.75 | 51.5 | 22.5 | 6.25 | 10.5 | 17.75 |
| Jul 2017 | 42.75 | 51.25 | 19.5 | 57.75 | 52.25 | 23.25 | 7.5 | 12.25 | 22.25 |
| Aug 2017 | 37 | 50.25 | 22 | 59 | 53 | 21 | 8.75 | 14 | 27.25 |
| Sep 2017 | 43.25 | 57 | 24.75 | 64.5 | 55.75 | 25.5 | 9 | 14 | 31.25 |
| Oct 2017 | 42 | 54 | 22.5 | 52 | 62 | 28.5 | 7.5 | 12.25 | 32 |
| Nov 2017 | 43 | 57.75 | 16.5 | 43 | 59.5 | 48.25 | 9.5 | 11.5 | 36.5 |
| Dec 2017 | 38.25 | 46.75 | 17 | 40.5 | 58.5 | 62.75 | 7.25 | 11.5 | 39.5 |
| Jan 2018 | 38 | 49 | 14.25 | 38.75 | 57.75 | 62.25 | 8.5 | 14 | 45.25 |
| Feb 2018 | 40.75 | 53 | 18 | 47.5 | 53 | 47 | 9.75 | 12.75 | 48.5 |
| Mar 2018 | 41.75 | 55 | 36.75 | 57.5 | 58 | 30.25 | 7 | 12.5 | 37.25 |
| Apr 2018 | 41.5 | 56 | 29.25 | 59.25 | 58.25 | 22.25 | 6.75 | 12 | 22 |
| May 2018 | 37.75 | 51.25 | 24.5 | 60 | 52 | 20.75 | 7 | 10.5 | 19.5 |
| Jun 2018 | 39.5 | 45.25 | 22 | 53.25 | 52.5 | 23 | 7.5 | 10.75 | 16 |
| Jul 2018 | 45.5 | 50.5 | 31.25 | 54.5 | 59 | 24.75 | 6.5 | 13.75 | 19.5 |
| Aug 2018 | 41.25 | 47.25 | 19.5 | 51 | 51.75 | 26.25 | 6.75 | 9 | 22 |
| Sep 2018 | 39 | 53.75 | 20.25 | 52.75 | 52.25 | 25.75 | 6.75 | 13.5 | 26.5 |
| Oct 2018 | 41 | 49 | 19.25 | 44 | 53.25 | 27 | 8.5 | 11 | 27.5 |
| Nov 2018 | 38.75 | 54.25 | 13.25 | 38.5 | 55.5 | 34.25 | 7.25 | 12.75 | 26.75 |
| Dec 2018 | 36.75 | 43.75 | 13 | 33 | 52 | 52.25 | 6.5 | 10.5 | 27 |
| Jan 2019 | 40.75 | 47.75 | 15.25 | 38 | 54.5 | 60.25 | 6.25 | 13.75 | 33 |
| Feb 2019 | 38 | 55 | 13.75 | 42.75 | 54.25 | 43.25 | 8.25 | 13.25 | 32.25 |
| Mar 2019 | 39 | 52.75 | 14 | 52 | 56.25 | 27 | 5 | 12 | 24.25 |
| Apr 2019 | 33.25 | 47.5 | 11.75 | 60.75 | 56 | 20.5 | 6 | 10.75 | 18.75 |
| May 2019 | 35.25 | 50.25 | 13.75 | 65.25 | 55.75 | 21.5 | 7.75 | 11 | 15.5 |
| Jun 2019 | 36.5 | 46.75 | 15.75 | 64.75 | 56.25 | 21.5 | 7.5 | 11.5 | 16.75 |
| Jul 2019 | 40.25 | 52.75 | 23.75 | 65.5 | 57.75 | 23.25 | 8.5 | 11.75 | 22.25 |
| Aug 2019 | 35.25 | 59 | 26.5 | 66 | 58.75 | 24.5 | 10 | 12.25 | 28.5 |
| Sep 2019 | 40.25 | 61 | 26.5 | 60.25 | 58.5 | 24 | 9.5 | 12.5 | 30 |
| Oct 2019 | 35.75 | 55.75 | 22.75 | 46.75 | 57.5 | 27 | 9.75 | 13.75 | 32 |
| Nov 2019 | 38.25 | 61 | 16.5 | 47 | 63.5 | 38.75 | 9.5 | 12.25 | 38.5 |
| Dec 2019 | 33.75 | 53 | 13.75 | 38.75 | 57.25 | 57.75 | 7.75 | 14.5 | 36.25 |
| Avg Yearly RSV | 43.53 | 52.67 | 17.89 | 52.89 | 54.73 | 35.11 | 7.68 | 11.4 | 26.43 |
