# Supplementary material for: Google trend analysis of the Indian population reveals a panel of seasonally sensitive comorbid symptoms with implications for monitoring the seasonally sensitive human population
Source: Popul Health Metr. 2024 Dec 30;22:40. doi: 10.1186/s12963-024-00349-7 (PMC11686857; doi:10.1186/s12963-024-00349-7)
Supplement: Supplementary file 3 — Additional file 3. [file 12963_2024_349_MOESM3_ESM.doc]

Supplementary Table S2B. Low search volume SCLD symptoms Without benchmark in India for the period from Jan 2015 to 2019

| Month-Year | Cyanosis | Sleep deprivation | Hypergammaglobulinemia |
| --- | --- | --- | --- |
| Jan 2015 | 47 | 29 | 0 |
| Feb 2015 | 35.25 | 36.75 | 0 |
| Mar 2015 | 43.5 | 54.2 | 0 |
| Apr 2015 | 59.25 | 48.25 | 0 |
| May 2015 | 36 | 41 | 0 |
| Jun 2015 | 36.25 | 53.5 | 0 |
| July 2015 | 44.5 | 52.25 | 0 |
| Aug 2015 | 48.75 | 48 | 0 |
| Sep 2015 | 23 | 48.25 | 0 |
| Oct 2015 | 50 | 27.75 | 0 |
| Nov 2015 | 57.75 | 48.6 | 0 |
| Dec 2015 | 67.75 | 39 | 0 |
| Jan 2016 | 53.5 | 31.4 | 0 |
| Feb 2016 | 56.75 | 36 | 0 |
| Mar 2016 | 58.5 | 54.25 | 0 |
| Apr 2016 | 44 | 60.75 | 0 |
| May 2016 | 39 | 39.4 | 0 |
| Jun 2016 | 35.5 | 38.5 | 0 |
| July 2016 | 48.75 | 46 | 0 |
| Aug 2016 | 32.75 | 44 | 0 |
| Sep 2016 | 51 | 33.75 | 0 |
| Oct 2016 | 46.75 | 33.8 | 0 |
| Nov 2016 | 21.25 | 34.5 | 0 |
| Dec 2016 | 50.5 | 39 | 0 |
| Jan 2017 | 46.25 | 35.2 | 0 |
| Feb 2017 | 59.5 | 30.25 | 0 |
| Mar 2017 | 45.25 | 31.25 | 0 |
| Apr 2017 | 45.75 | 29.6 | 0 |
| May 2017 | 54.75 | 49 | 0 |
| Jun 2017 | 63.5 | 43 | 0 |
| July 2017 | 54.25 | 51.8 | 0 |
| Aug 2017 | 55.75 | 32.75 | 0 |
| Sep 2017 | 49.75 | 36.5 | 0 |
| Oct 2017 | 57 | 44.4 | 0 |
| Nov 2017 | 65 | 31.25 | 0 |
| Dec 2017 | 60 | 32.4 | 0 |
| Jan 2018 | 76.75 | 30.75 | 0 |
| Feb 2018 | 45 | 42 | 0 |
| Mar 2018 | 65 | 43 | 0 |
| Apr 2018 | 53 | 33.2 | 0 |
| May 2018 | 50.5 | 35.25 | 0 |
| Jun 2018 | 53.75 | 41.5 | 0 |
| July 2018 | 48.75 | 37.5 | 0 |
| Aug 2018 | 51.5 | 43.5 | 0 |
| Sep 2018 | 59.25 | 38.8 | 0 |
| Oct 2018 | 43.75 | 35.25 | 0 |
| Nov 2018 | 47 | 38.25 | 0 |
| Dec 2018 | 64.25 | 39.2 | 0 |
| Jan 2019 | 57.25 | 34.5 | 0 |
| Feb 2019 | 74.25 | 39.25 | 0 |
| Mar 2019 | 45 | 42.25 | 0 |
| Apr 2019 | 51.75 | 45.75 | 0 |
| May 2019 | 43 | 57.33 | 0 |
| Jun 2019 | 55 | 42.25 | 0 |
| July 2019 | 61.75 | 43.25 | 0 |
| Aug 2019 | 58.25 | 42.75 | 0 |
| Sep 2019 | 67.5 | 45.6 | 0 |
| Oct 2019 | 49.5 | 42.25 | 0 |
| Nov 2019 | 67.5 | 47 | 0 |
| Dec 2019 | 69 | 39.4 | 0 |
| **Avg yearly RSV** | **51.7** | **40.75** | **0** |
