# Supplementary material for: Google trend analysis of the Indian population reveals a panel of seasonally sensitive comorbid symptoms with implications for monitoring the seasonally sensitive human population
Source: Popul Health Metr. 2024 Dec 30;22:40. doi: 10.1186/s12963-024-00349-7 (PMC11686857; doi:10.1186/s12963-024-00349-7)
Supplement: Supplementary file 4 — Additional file 4. [file 12963_2024_349_MOESM4_ESM.doc]

Supplementary Table S3. Pubmed literature supports the association of SCLD symptoms with various diseases.

| **SCLD symptoms** | **Disease Class** | **Associated Diseases** | **Pubmed ID** |
| --- | --- | --- | --- |
| **Fatigue** | **Non-communicable diseases** | Cancer | PMID: 32025928, PMID: 25113839 |
|  | **Communicable diseases** | HIV/AIDS | PMID: 31815724 |
|  |  | COVID | PMID: 36215063 |
|  |  | Neuromuscular disorder | PMID: 20111846 |
|  | **Neurological disorders** | Stroke | PMID: 23339207 |
|  |  | Parkinson's |
|  |  | Alzheimer's |
|  |  | Myasthenia gravis |
|  |  | Traumatic brain injury |
|  |  | Skin diseases | PMID: 33196099 |
|  |  |  |  |
| **Shortness of breath** | **Non-communicable diseases** | COPD | PMID: 36429887 |
|  |  | Asthma | PMID: 36682372 |
|  |  | Oropharyngeal cancer | PMID: 36640945 |
|  |  | Recurrent laryngeal papillomatosis | PMID: 36477391 |
|  |  | Pneumocystis pneumonia | PMID: 33759428,PMID: 31654993 |
|  |  | Hepatocellular carcinoma (HCC) | PMID: 24956436 |
|  | **Communicable diseases** | COVID -19 | PMID: 35568052, PMID: 35664995 |
|  |  | Severe acute respiratory syndrome (SARS) | PMID: 15021055 |
|  | **Neurological disorders** | Parkinson's disease | PMID: 32419523 |
|  |  | Stiff person syndrome | PMID: 31400804 |
|  |  | Myotonic dystrophy | PMID: 36669462 |
|  |  |  |  |
| **Fever** | **Non-communicable diseases** | Osteomyelitis | PMID: 33563770 |
|  |  | Cancer | PMID: 28441374 |
|  |  | Sinusitis | PMID: 25724501 |
|  | **Communicable diseases** | HIV | (PMID: 8698988) |
|  |  | Yellow fever | PMID: 36404585, PMID: 28834938 |
|  |  | Allergic rhinitis |
|  |  | Streptococcal throat infection |
|  |  | Tuberculosis (TB) | PMID: 26198113 |
|  |  | UTI | PMID: 28087935, PMID: 30592257 |
|  | **Neurological disorders** | Otitis media | PMID: 29445883 |
|  |  | Bacterial meningitis | PMID: 20445424, PMID: 2591401 |
|  |  | Encephalitis | PMID: 10517930 |
|  |  |  |  |
| **Obesity** | **Non-communicable diseases** | Obstructive sleep apnea | PMID: 34697666 |
|  |  | Cellulitis |
|  |  | Pancreatitis |
|  |  | Flu-related pneumonia | PMID: 35058571 |
|  | **Communicable diseases** | UTI | PMID: 35058571 |
|  | **Neurological disorders** | Parkinson's disease and Alzheimer's disease | PMID: 29257910 |
|  |  | Multiple sclerosis | PMID: 34207197, PMID: 36457996 |
|  |  | ADHD | PMID: 25665976 |
|  |  |  |  |
| **Hypergamma globulinemia** | **Non-communicable diseases** | Multiple myeloma | PMID: 15335188 |
|  |  | Gaucher's disease | PMID: 8576322 |
|  |  | Chronic liver disease | https://www.sciencedirect.com/referencework/9780122267659/encyclopedia-of-immunology |
|  |  | Rheumatoid arthritis |
|  |  | Sjögren's syndrome |
|  |  | SLE |
|  |  | Cystic fibrosis |
|  | **Communicable diseases** | HIV/AIDS | PMID: 14604962 |
|  |  | Syphilis | PMID: 21674575 |
|  |  | Tuberculosis | https://www.sciencedirect.com/referencework/9780122267659/encyclopedia-of-immunology |
|  | **Neurological disorders** | Hypertrophic pachymeningitis | PMID: 20844883 |
|  |  |  |  |
| **Cyanosis** | **Non-communicable diseases** | Pulmonary hypertension | PMID: 32644593 |
|  |  | COPD |
|  |  | Obstructive sleep apnea |
|  |  | Congenital heart diseases |
|  |  | Deep vein thrombosis |
|  |  | Multiple myelomas, Polycythemia, and Macroglobulinemia |
|  | **Communicable diseases** | Pertussis | PMID: 7761715 |
|  |  | Secondary syphilis | PMID: 8179446 |
|  |  | Neonatal herpes | PMID: 2537945 |
|  | **Neurological disorders** | Cerebral palsy | PMID: 16087059 |
|  |  | Methemoglobinemia type 2 | PMID: 34267908 |
|  |  |  |  |
| **Headache** | **Non-communicable diseases** | Restless legs syndrome (RLS) | PMID: 29539610 |
|  |  | Bruxism |
|  |  | Ischaemic disease |
|  |  | Primary sleep disorders | PMID: 12505479 |
|  |  | Coeliac disease | PMID: 30301194 |
|  | **Communicable diseases** | Meningitis | PMID: 37867184 |
|  | **Neurological disorders** | Systemic lupus erythematosus | PMID: 24527723 |
|  |  | Systemic vasculitis |
|  |  | Central nervous system (CNS) vasculitis |
|  |  | Neurocutaneous disorders | PMID: 36066692 |
|  |  | Meningitis | PMID: 37867184 |
|  |  |  |  |
| **Snoring** | **Non-communicable diseases** | Obstructive sleep apnoea | PMID: 33141943 |
|  |  | Sleep disorders | PMID: 29878472 |
|  |  | Non-alcoholic fatty liver | PMID: 32518245 |
|  |  | Cardiovascular diseases | PMID: 34029563 |
|  |  | Cystic fibrosis | PMID: 32841845 |
|  | **Communicable diseases** | HIV | PMID: 32861059 |
|  | **Neurological disorders** | Parkinson's disease | PMID: 29042002 |
|  |  | Active convulsive epilepsy | PMID: 24582322 |
|  |  |  |  |
| **Sweating** | **Non-communicable diseases** | Mood disorders | PMID: 32996756 |
|  |  | Gastroesophageal reflux disease |
|  |  | Hyperthyroidism |
|  | **Communicable diseases** | SARS | PMCID: PMC7095307 |
|  |  | Tuberculosis | PMID: 35583175 |
|  | **Neurological disorders** | Peripheral neuropathies | PMID: 30459040 |
|  |  | Hyperhidrosis | PMID: 36627476 |
|  |  |  |  |
| **Edema** | **Non-communicable diseases** | Liver cirrhosis | https://www.informedhealth.org/ |
|  |  | Kidney dysfunction |
|  |  | Congestive heart failure |
|  | **Communicable diseases** | Mumps | PMID: 36277528, PMID: 36277528 |
|  |  | Bordetella pertussis | PMID: 19367413 |
|  | **Neurological disorders** | Bacterial meningitis | PMID: 2498090 |
|  |  | Encephalitis | PMID: 26852357 |
|  |  |  |  |
| **Sleep deprivation** | **Non-communicable diseases** | Obstructive lung disease | PMID: 19201722 |
|  |  | Restrictive lung disease |
|  |  | Gastroesophageal reflux disease |
|  |  | COPD |
|  |  | Fibromyalgia |
|  |  | Chronic renal disease |
|  |  | Encephalopathy | PMID: 25315420 |
|  | **Communicable diseases** | HIV/AIDS | PMID: 35614470 |
|  |  | Tuberculous meningitis | PMID: 18294655 |
|  | **Neurological disorders** | Parkinson's disease | PMID: 33896849 |
|  |  | Alzheimer's disease | PMID: 11602004 |
|  |  |  |  |
| **Cough** | **Non-communicable diseases** | Bronchiectasis | PMID: 28602999 |
|  |  | Gastroesophageal reflux disease (GERD) | PMID: 32228010 |
|  |  | Chronic obstructive pulmonary disease (COPD) |
|  |  | Rhinosinusitis |
|  | **Communicable diseases** | Pertussis | PMID: 30321509, PMID: 23287746 |
|  |  | Influenza | PMID: 29083802, PMID: 33760547 |
|  |  | Tuberculosis | PMID: 15623010 |
