# Supplementary material for: Google trend analysis of the Indian population reveals a panel of seasonally sensitive comorbid symptoms with implications for monitoring the seasonally sensitive human population
Source: Popul Health Metr. 2024 Dec 30;22:40. doi: 10.1186/s12963-024-00349-7 (PMC11686857; doi:10.1186/s12963-024-00349-7)
Supplement: Supplementary file 5 — Additional file 5. [file 12963_2024_349_MOESM5_ESM.doc]

Supplementary Table S4. RSV data of October 2023 from India to evident the symptoms prioritization changed in accordance with environmental changes.

| Day | Sleep deprivation | Fatigue | Snoring | Dry cough | Cyanosis | Sweating | Edema | High fever | Severe headache | Shortness of breath | Obesity | Hypergammaglobulinemia |
| --- | --- | --- | --- | --- | --- | --- | --- | --- | --- | --- | --- | --- |
| 01-10-2023 | 46 | 89 | 75 | 82 | 23 | 89 | 64 | 67 | 90 | 90 | 58 | 41 |
| 02-10-2023 | 46 | 91 | 67 | 75 | 42 | 99 | 72 | 85 | 100 | 45 | 81 | 27 |
| 03-10-2023 | 42 | 86 | 70 | 90 | 70 | 60 | 66 | 63 | 85 | 73 | 82 | 0 |
| 04-10-2023 | 38 | 100 | 85 | 86 | 48 | 97 | 82 | 91 | 65 | 94 | 100 | 0 |
| 05-10-2023 | 58 | 86 | 64 | 77 | 40 | 77 | 71 | 73 | 54 | 58 | 75 | 0 |
| 06-10-2023 | 43 | 83 | 66 | 75 | 55 | 63 | 88 | 75 | 85 | 79 | 78 | 0 |
| 07-10-2023 | 54 | 66 | 51 | 85 | 32 | 85 | 64 | 87 | 18 | 44 | 75 | 0 |
| 08-10-2023 | 55 | 71 | 58 | 79 | 56 | 94 | 58 | 83 | 44 | 75 | 68 | 0 |
| 09-10-2023 | 26 | 90 | 52 | 83 | 46 | 91 | 93 | 100 | 61 | 85 | 80 | 0 |
| 10-10-2023 | 48 | 98 | 64 | 83 | 59 | 100 | 85 | 89 | 59 | 58 | 81 | 0 |
| 11-10-2023 | 28 | 82 | 67 | 94 | 55 | 87 | 74 | 75 | 86 | 54 | 92 | 0 |
| 12-10-2023 | 0 | 93 | 72 | 89 | 27 | 86 | 91 | 71 | 91 | 46 | 83 | 32 |
| 13-10-2023 | 0 | 78 | 84 | 86 | 41 | 97 | 71 | 70 | 36 | 64 | 67 | 0 |
| 14-10-2023 | 16 | 70 | 46 | 66 | 53 | 83 | 63 | 64 | 68 | 69 | 54 | 0 |
| 15-10-2023 | 32 | 77 | 60 | 89 | 53 | 78 | 67 | 87 | 61 | 68 | 76 | 100 |
| 16-10-2023 | 0 | 78 | 66 | 100 | 9 | 72 | 76 | 80 | 42 | 65 | 82 | 0 |
| 17-10-2023 | 24 | 78 | 50 | 86 | 100 | 49 | 100 | 78 | 55 | 81 | 72 | 30 |
| 18-10-2023 | 27 | 69 | 82 | 77 | 43 | 56 | 97 | 71 | 75 | 79 | 88 | 0 |
| 19-10-2023 | 0 | 68 | 69 | 73 | 58 | 54 | 71 | 60 | 58 | 70 | 79 | 43 |
| 20-10-2023 | 0 | 67 | 44 | 80 | 50 | 56 | 53 | 66 | 71 | 89 | 88 | 0 |
| 21-10-2023 | 0 | 67 | 81 | 78 | 0 | 62 | 69 | 59 | 56 | 58 | 55 | 0 |
| 22-10-2023 | 47 | 56 | 72 | 73 | 25 | 63 | 53 | 78 | 75 | 76 | 45 | 0 |
| 23-10-2023 | 0 | 56 | 76 | 83 | 28 | 67 | 59 | 51 | 100 | 83 | 51 | 0 |
| 24-10-2023 | 56 | 71 | 99 | 92 | 41 | 73 | 64 | 74 | 86 | 69 | 62 | 0 |
| 25-10-2023 | 32 | 85 | 62 | 94 | 35 | 68 | 86 | 80 | 65 | 70 | 79 | 0 |
| 26-10-2023 | 39 | 63 | 74 | 90 | 42 | 59 | 94 | 80 | 53 | 69 | 77 | 0 |
| 27-10-2023 | 0 | 69 | 29 | 91 | 61 | 57 | 78 | 65 | 86 | 48 | 66 | 0 |
| 28-10-2023 | 40 | 60 | 100 | 89 | 57 | 72 | 77 | 86 | 76 | 63 | 69 | 0 |
| 29-10-2023 | 100 | 62 | 71 | 86 | 40 | 58 | 50 | 66 | 48 | 49 | 67 | 0 |
| 30-10-2023 | 36 | 75 | 78 | 89 | 42 | 61 | 83 | 66 | 81 | 62 | 80 | 0 |
| 31-10-2023 | 20 | 72 | 94 | 87 | 37 | 61 | 70 | 71 | 16 | 100 | 77 | 0 |
| Avg RSV of month | 30.74 | 76 | 68.65 | 77 | 44.13 | 78 | 73.84 | 79 | 66 | 80 | 73.77 | 8.80 |
